# Supplementary material for: Functional Analysis of a Multiple-Domain CTL15 in the Innate Immunity, Eclosion, and Reproduction of Tribolium castaneum
Source: Cells. 2023 Feb 13;12(4):608. doi: 10.3390/cells12040608 (PMC9954269; doi:10.3390/cells12040608)
Supplement: Supplementary file 1 [file cells-12-00608-s001.zip › cells-2159909-supplementary.pdf]

Supplmental figures and tables

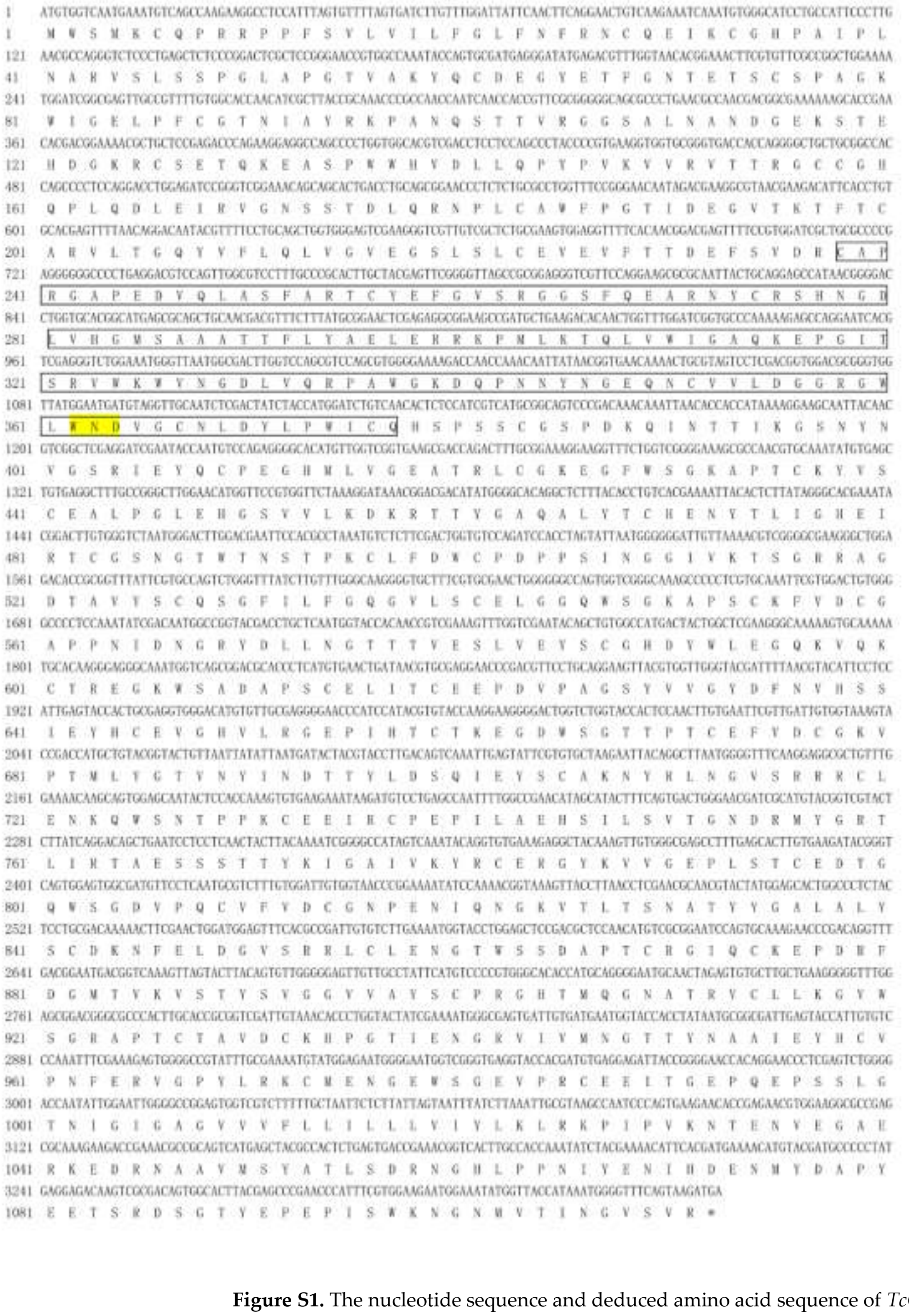

Figure S1. The nucleotide sequence and deduced amino acid sequence of *TcCTL15*. The amino acids and nucleotides were numbered at left. The black open box indicated the carbohydrate recognition domains (CRD) predicted by SMART and the highlight in yellow indicated the motifs with putative carbohydrate binding specificity.

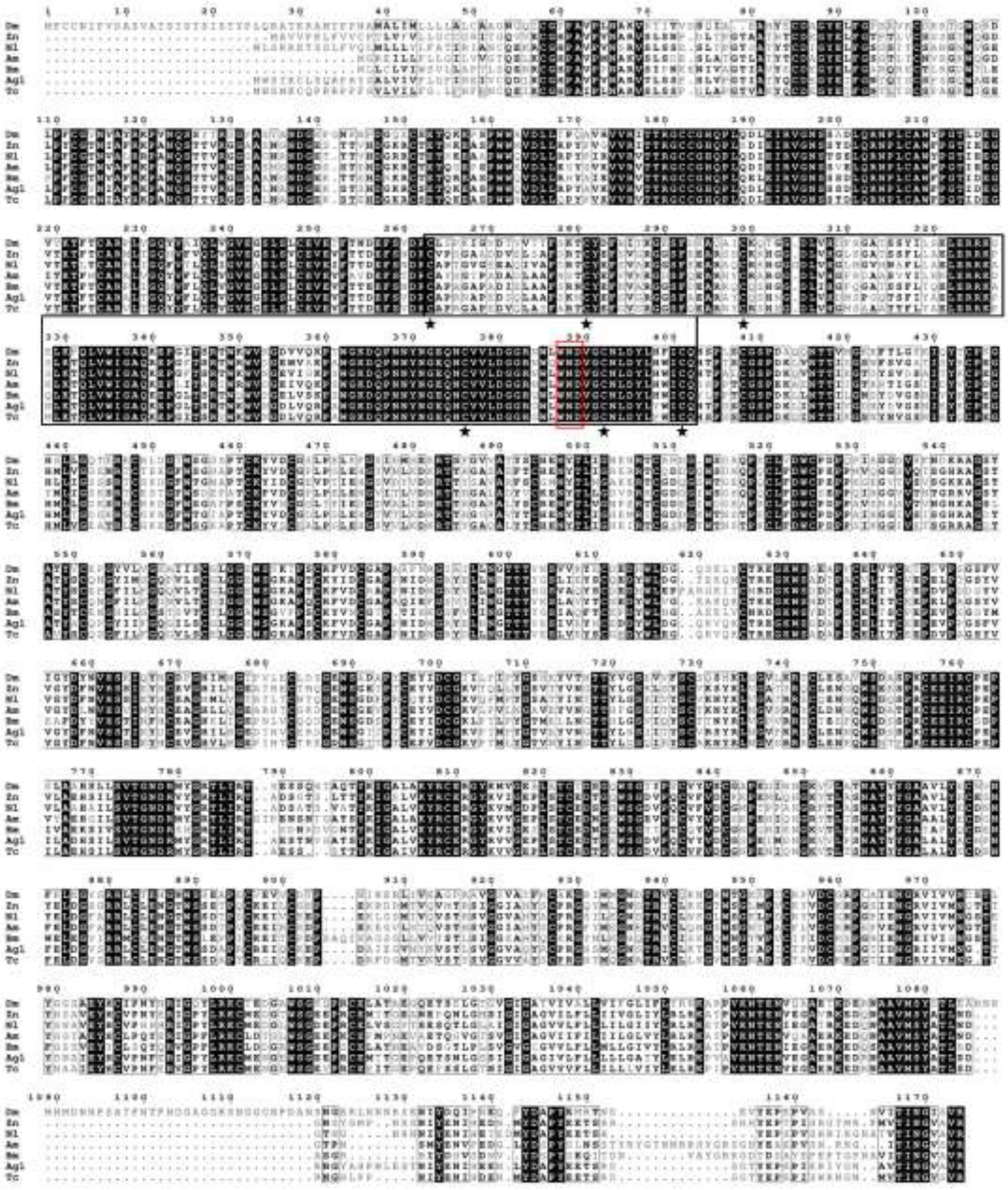

Figure S2. Multiple sequence alignment of TcCTL15 with homologues from other model insects. Dm: *Drosophila melanogaster*, Zn: *Zootermopsis nevadensis*, Nl: *Nilaparvata lugens*, Am: *Apis mellifera*, Bm *Bombyx mori*, Agl: *Anoplophora glabripennis* and Tc: *Tribolium castaneum*. Amino acids involved carbohydrate recognition domains (CRDs) in sugar recognition were boxed with black. The WND motif was frammed in red. Conserved cysteine is denoted by black asterisks. Highly similar amino acids were shaded in black background.

Table S1 Oligonucleotide primers used in the current study.

Name	Purpose	Sequence (5′-3′)
TcCTL15-F	Clone	atgggtcgcggatccgaattcTGCGCCCCGAGGGGGGCC
TcCTL15-R		gtggtggtggtggtgctcgagTTGACAGATCCATGGTAGATAGTCG
ds-*TcCTL15*-F	RNAi	taatacgactcactatagggAGTGCGATGAGGGATATGAGAC
ds-*TcCTL15*-R		taatacgactcactatagggGTTAAAACTCGTGCACAGGTGA
ds-*TcVer*-F		taatacgactcactatagggGTCTTGGTGGACCAAG
ds-*TcVer*-R		taatacgactcactatagggCCGCCATTTCGTGATC
TcRPS3-qF	qRT-PCR	TCAAATTGATCGGAGGTTTG
TcRPS3-qR		GTCCCACGGCAACATAATCT
TcCTL15-qF		AAGCCGATGCTGAAGACACA
TcCTL15-qR		AATTTGTTTGTCGGGACTGCC
Dif1-qF		GCCCGATGAGAGCATCAACAT
Dif1-qR		CTTGTTGGGGTTGTTGTTGGAT
Dif2-qF		CAAGTCACGCAATGTCTCTAGA
Dif2-qR		ATCGGTTCGAGTTCGATTTGGT
Rel-qF		GAGCCTGTGCAGATTGATATG
Rel-qR		CTGGGGCAACATCGGTAAACA
STAT-qF		TTCGTACGTGAGGCAGTGTC
STAT-qR		TTCGTCTCAAATCGTCGGCA
att1-qF		GCCAACTGGGATACTCCCATA
att1-qR		TACCATTCCTCCATAAATTCCCA
att2-qF		TCGTAATTTTTGCACTCGCC
att2-qR		TGGGGCATCTTTATTGACGAA
att3-qF		TTTCCCAACTCTGGCGTCC
att3-qR		TGCCATTGGGTCATTATTGAG
def1-qF		CACAGCCTTTCCAGCAGATG
def1-qR		CAAATAGAGACAATGAGCGGCA
def2-qF		GCTACTCATTGTCGCTCTAGTT
def2-qR		AAAGAGGCAATGGGTCGCAC
def3-qF		TGTTTGCAATCACTGCTTACCC
def3-qR		TGTTGCAATAGCCTCCACGTT
col1-qF		TTGCCAGCCGAAGAGTACAA
col1-qR		GTCTTTCTCGGTGGAGTTCA
cecr2-qF		TCCTGTAGTAAAAGACGGAGT
cecr2-qR		CAGTATCCATAGTTGTGTACG
cecr3-qF		TGTTCCCGTGGTTAAAAATGG
cecr3-qR		ATACTGAATTGGGATTCCTCC
Vg-qF		ATGAGTTGTTCTGAAGTTATGTA
Vg-qR		AACTGGGCTGCGAGAGAGA
FOXO-qF		TCAGACCTCGAGCTTCCTCA
FOXO-qR		TGCAAGCTGATCGGGTGAAT
JHAMT-qF		CATCTCGCCCTATCACCATTCG
JHAMT-qR		CCGCTGAAACCGATTTTGACAA
FDH-qF		TCCTGGTGAACAATGCTGGG
FDH-qR		TTGTGGCAACACACACAGGGAA
spook-qF		ATGTTTCAGTTTCGTGGTTTTAGC
spook-qR		TGTGATTACAGCCGCAATCTTAC
shadow-qF		TAAGAGCCCGCAAATCCAG
shadow-qR		CGTCACGCCCGCTTGTAT
shade-qF		TACTTCGGCTTCCCCTTCTG
shade-qR		GTGAATCCCCGCAGATATGAA
E74-qF		CTACCTTCTCCAGCAGCACCAC
E74-qR		AGTGGCCGCCTGGATCA
Ftz-F1-qF		TAGACAAAACGCAAAGGAAAAGG
Ftz-F1-qR		GTTTGTACATCGGACCGAATTT
Br-C-qF		CACAACACTTCTGTCTGCGGTG
Br-C-qR		CACAACACTTCTGTCTGCGGTG

CCAPR1-qF	ATGACACTAGTTTCGCTCTC
CCAPR1-qR	TGAGTGGTGCTAAACTTTGG
CCAPR2-qF	TTCTACAAGACCGAGCAGTTCAC
CCAPR2-qR	CAAGCCACCGTTATCCTCCA
Rickets-qF	TTTACCGTGCCAGGACCTG
Rickets-qR	CAGAATCGCGTACAAGAACG
